# The lactate dehydrogenase gene is involved in the growth and metabolism of *Lacticaseibacillus paracasei* and the production of fermented milk flavor substances

**DOI:** 10.3389/fmicb.2023.1195360

**Published:** 2023-06-09

**Authors:** Sichang Fang, Xin Song, Liru Cui, Jinping Bai, Han Lu, Shijie Wang

**Affiliations:** ^1^College of Food Science and Biology, Hebei University of Science and Technology, Shijiazhuang, Hebei, China; ^2^School of Health Science and Engineering, University of Shanghai for Science and Technology (USST), Shanghai, China

**Keywords:** *Lacticaseibacillus paracasei*, lactate dehydrogenase, CRISPR, fermented milk, metabolomics

## Abstract

**Objective:**

Lactate dehydrogenase (ldh) in lactic acid bacteria is an important enzyme that is involved in the process of milk fermentation. This study aimed to explore the changes and effects of fermented milk metabolites in mutant strains after knocking out the *ldh* gene of *Lacticaseibacillus paracasei*.

**Methods:**

The *ldh* mutant Δ*AF91_07315* was obtained from *L. paracasei* using clustered regularly interspaced short palindromic repeats technology, and we determined fermented milk pH, titratable acidity, viable count, and differential metabolites in the different stages of milk fermentation that were identified using metabolomic analysis.

**Results:**

The results showed that the growth rate and acidification ability of the mutant strain were lower than those of the wild-type strain before the end of fermentation, and analysis of the differential metabolites showed that lactate, L-cysteine, proline, and intermediate metabolites of phenylalanine, tryptophan, and methionine were downregulated (*P* < 0.05), which affected the growth initiation rate and acidification ability of the strain. At the end of fermentation (pH 4.5), the fermentation time of the mutant strain was prolonged and all differential metabolites were upregulated (*P* < 0.05), including amino acids and precursors, acetyl coenzyme A, and other metabolites involved in amino acid and fatty acid synthesis, which are associated with the regulation of fermented milk flavors. In addition, riboflavin was upregulated to promote the growth of the strain and compensate for the growth defects caused by the mutation.

**Conclusion:**

Our data established a link between the *AF91_07315* gene and strain growth and metabolism and provided a target for the regulation of fermented milk flavor substances.

## 1. Introduction

Fermented milk is a nutrient product made from milk co-cultured with a starter culture and is widely accepted for its unique taste and flavor (Wang et al., 2021c). Lactic acid bacteria (LAB) are a traditional starter culture that uses milk as a means to produce nutrients and flavored substances. These substances include organic acid, peptides, amino acids, and short-chain fatty acids, which improve the digestion and bioavailability of milk components, inhibit harmful bacteria in the gastrointestinal tract, prevent cancer, reduce lactose intolerance, and lower cholesterol levels (Shiby and Mishra, 2013). Organic acid is the main component of fermented milk, and lactic acid enhances the flavor of fermented milk and maintains probiotics in the human gastrointestinal gut, which is beneficial against septic and bacterial infections, stimulates intestinal motility, and enhances the immune response to antigenic invasion (Shukla et al., 2008; Vieco-Saiz et al., 2019; Mathur et al., 2020). However, the mechanism of metabolic regulation by LAB in fermented milk is unclear, and understanding LAB metabolic pathways can help regulate the production of flavored substances in fermented milk.

Lactate dehydrogenase, a key enzyme in the glycolytic pathway for lactate production, uses pyruvate as an electron acceptor, accompanied by the oxidation of nicotinamide adenine dinucleotide, to catalyze the pyruvate reaction to produce lactate (Kandler, 1983). To date, genetic engineering has been applied to knockout and overexpress *ldh* to change the energy flow and achieve high industrial lactate production (Singhvi et al., 2018; Bleckwedel et al., 2020). Furthermore, changes in the flavor of fermented milk caused by *ldh* gene deletion have been investigated. This study showed that lactic acid production was eliminated by knocking out the *ldh* gene in *Thermoanaerobacterium saccharolyticum* JW/SL-YS485, thereby increasing acetic acid and ethanol production (Desai et al., 2004). In addition, the elimination of *ldhD* using the double cross strategy increased the production of acetaldehyde, diacetyl, and acetoin in *Lactobacillus johnsonii* (Lapierre et al., 1999). These flavored substances have only been characterized in the medium; however, few studies have investigated the role of lactate dehydrogenase in fermented milk, and effective characterization of its metabolic components during fermentation is important for improving its flavor.

Mass spectrometry-based untargeted metabolomics is a highly specific and sensitive technology for monitoring small molecular substrates produced by microorganisms, and it screens samples for biomarkers or differential substances and helps identify differential metabolites that modulate phenotypes (Johnson et al., 2016; Guijas et al., 2018). Research on milk metabolomics has mainly focused on the effect of *Streptococcus thermophilus* (*S. thermophilus*), co-cultured with lactobacilli on the fermentation process and quality (Tomassini et al., 2019; Li et al., 2021), and there are few metabolic studies on single-strain fermented milk. We used *L. paracasei* CGMCC4691 as a test bacterium owing to its beneficial characteristics. As a co-fermenter of fermented milk, it can enhance immunity, improve intestinal metabolites, and regulate intestinal flora. In addition, the inactivated strain can improve the brain–intestinal axis and reduce continuous damage to brain nerves (Miao et al., 2022; Zhang et al., 2022). In this study, the mutant strain Δ*AF91_07315* was generated from *L. paracasei* using the clustered regularly interspaced short palindromic repeats (CRISPR) technology, and pH, titratable acidity (TA), and bacterial counts were compared between the wild-type (WT) and Δ*AF91_07315* strains. In addition, differential metabolites were identified using non-targeted metabolomics to assess the metabolic role of Δ*AF91_07315* and provide targets for the modulation of flavored substances.

## 2. Materials and methods

### 2.1. Strains, culture media, and growth conditions

*L. paracasei* CGMCC4691 was obtained from our laboratory. The pLL plasmid was provided by the University of Shanghai for Science and Technology. *E. coli* Top10 competent cell was purchased from TianGen Biotech. *L. paracasei* CGMCC4691 and Δ*AF91_07315* mutants were cultured in De Man Rogosa Sharpe (MRS) medium at 37 °C for 14–16 h for skim milk fermentation. *E. coli* Top10 was used for cloning and was grown on Luria Bertani medium at 30 °C for 12–14 h. When needed, a suitable antibiotic was supplemented at 10 μg/ml for Δ*AF91_07315* and 50 μg/ml for *E. coli* Top10.

### 2.2. Construction of *L. paracasei* CGMCC4691 Δ*AF91_07315*

#### 2.2.1. Construction of knockout plasmids

The knockout plasmid was constructed using polymerase chain reaction (PCR) and seamless cloning technology. The primers used in this study are presented in Table 1. First, the vector skeleton was generated using an *Apa*I-*Xba*I (Vazyme Biotech Co., Ltd.)-digested pLL plasmid. Three fragments, 1,300 bp homologous arms upstream and downstream (obtained by PCR using primers up/down-F/R with *L. paracasei* CGMCC4691 genome as a template), and sgRNA (obtained by PCR using primers sgRNA-F/R with pLL as a template) were generated and purified using a gel purification kit (Axygen, SV, USA), all of which were connected to generate up–down-sgRNA fragments. The resulting fragment was ligated into the vector skeleton to produce pLL-07315 using a seamless cloning kit (Vazyme Biotech Co., Ltd.) and verified using ldhYZ-F/R following transformation into *E. coli* Top10 competent cells.

**Table 1:** Primer sequences used in this study.

**Primer**	**Sequence(5′-3′)**
Up-F	TTTTCTAAACTAGGGCCCCACTACTGGCCGCCTAC
Up-R	TGGAGGGGAAGGGTTTTCTTATGCCTATCCACTCGACATTGAC
Down-F	TCGAGTGGATAGGCATAAGAAAACCCTTCCCCTCCACT
Down-R	GTCGGTGCTTTTTTTGAGACACGATTATGGGCACGG
Sgrna-F	CCGTGCCCATAATCGTGTCTCAAAAAAAGCACCGACTCG
Sgrna-R	CATGAGGAGGAATTTGAGTCTAGAGACGCATCTGATGGATGTAG GTTTTAGAGCTAGAAATAGCAAGTTAAAATAAGGC
deleteYZ-R	CCATCAAAAGCTTTGATCAACGC
deleteYZ-F	CAATGATCGCAATGTTGCGAATAT
Ldhyz-F	CTATCAACACACTCTTAAGTTTGCTTCTAAG
Ldhyz-R	AGTTTTGAGGCAAAATTTTTGAGTGACA

#### 2.2.2. Deletion of the AF91_07315 gene

A competent cell of *L. paracasei* CGMCC4691 was prepared as described by Song et al. (2017). The constructed plasmid was electroporated into cells using a GenePulser Xcell (Bio-Rad) and a 2 mm cuvette (BTX) at 2.0 KV, 200Ω, and 25 μF. After recovery for 3 h in the MRS medium, the cells were separated on agar plates and grown for 72 h. A single strain was selected and verified by PCR and named pLL: 07315. Precise sequencing was also performed using BGI.

#### 2.2.3. Fermented milk preparation

Skim milk (10% w/v) was prepared from skim milk powder (Shijiazhuang Junlebao Dairy Co., Ltd.). Skim milk powder was dissolved in 50°C sterile water and autoclaved at 115 °C for 15 min. After cooling to 37 °C, 400 ml of milk was transferred into six different sterile flasks. Pre-cultured *L. paracasei* CGMCC4691 and its mutants were inoculated in triplicate at a 5% inoculum size (2×10^7^ CFU). The mixed milk was fermented until the pH reached an end-point of 4.5. Subsequently, a sample from each flask was collected before (4 h) and after (8 h) the fermentation periods and end-points. Before analysis, these samples were stored at −80 °C.

### 2.3. TA and bacterial count determination

#### 2.3.1. Determination of pH and TA

pH was measured using a pH meter (METTLER TOLEDO, China). TA was determined using 0.1 mol/L NaOH with phenolphthalein as a color indicator, and the result was expressed as °T.

#### 2.3.2. Determination of viable count

The viable counts of different periods during milk fermentation were determined as follows: 1 ml of the sample was diluted in 9 ml of normal saline (0.85%) by vortex oscillation, and the dilution was performed continuously. The diluted liquid (100 μl) was cultured using the MRS agar plate pouring method. The viable counts were calculated after incubation at 37 °C for 48 h and expressed as CFU/ml.

### 2.4. UPLC-Q-TOF MS^E^ analysis

#### 2.4.1. Sample pretreatment

The fermented milk samples stored at −80 °C were thawed. We transferred 50 mg of each sample into a fresh centrifuge tube, and 400 μl methanol solution (containing 5 μg/ml L-2-chloro-phenylalanine as the internal standard) was added and vortexed for 1 min. The sample was then mixed two times at 60 Hz for 3 min and centrifuged at 13,000 rpm at 4°C for 10 min using a cryogenic centrifuge (Thermofisher FRESCO21, USA). Supernatants were collected for analysis. Equal volumes of all samples were mixed as quality control samples. All reagents were analytically pure and purchased from Shanghai.

#### 2.4.2. Ultra performance liquid chromatography analysis

The samples were analyzed on an Agilent 1290 infinity (USA), incorporating a HSS T3 chromatographic column (water, 2.5 μm, 100^*^2.1 mm). The mobile phases A and B consisted of water and acetonitrile (mixed with 0.1% formic acid). The flow rate of the mobile phase was 0.4 ml/min at a column temperature of 40°C. The injection volume was 4 μl. The optimized gradient elution was 0–3 min, 20% B; 3–9 min, 20–95% B; 9–13 min, 95% B; 13–13.1 min, 95–5% B; and 13.1–16 min, 5% B.

#### 2.4.3. Mass spectrometry (MS) analysis

Mass spectrometry (MS) analysis was performed using a Q-TOF-MS^E^ (Agilent 6545 UHD, USA), coupled with positive (ESI+) and negative (ESI–) modes. The capillary voltage was 4.5 kV and 3.5 kV in ESI+ and ESI–, respectively, and the drying gas flow was 8 L/min and 10 L/min at a temperature of 325°C, respectively. The nebulizer voltage was set at 20 psi. The fragmentation voltage was set to 120 V. The MS scans were performed over an entire information range of 50–1,500 m/z.

### 2.5. Statistical analysis

Fermentation was performed in triplicate for the starter culture. Metabolic data were collected using Agilent MassHunter Qualitative Analysis B.0 8.0 software. A series of procedures were performed using the R software XCMS package, including peak identification, retention time correction, and automatic integration. The chromatographic peak values were normalized by dividing the original peak area by the internal standard peak. Differences in metabolic profiles and visual differences were achieved using R-script, such as principal component analysis and orthogonal projections to latent structures discriminant analysis (OPLS-DA).

Differential metabolites were identified using the OPLS-DA model, variable importance in the projection (VIP ≥1), independent sample *t*-test (*P* < 0.05), and fold change (FC ≥1.1 or ≤0.9). The differential metabolites of interest were identified by matching accurate molecular weights against the Kyoto Encyclopedia of Genes and Genomes (KEGG; https://www.kegg.jp) online database using the following qualitative methods: the adduct ions in ESI+ mode included [M+H]+ and [M+Na+]+ and those in ESI– mode included [M–H] –; the quality error value was selected as 30 ppm. Thereafter, the metabolic pathway was matched against KEGG using MetaboAnalyst software, and the metabolic effects were assessed by metabolic enrichment analysis.

## 3. Results

### 3.1. Construction of *L. paracasei* CGMCC4691Δ*AF91_07315*

**Figure 1:**
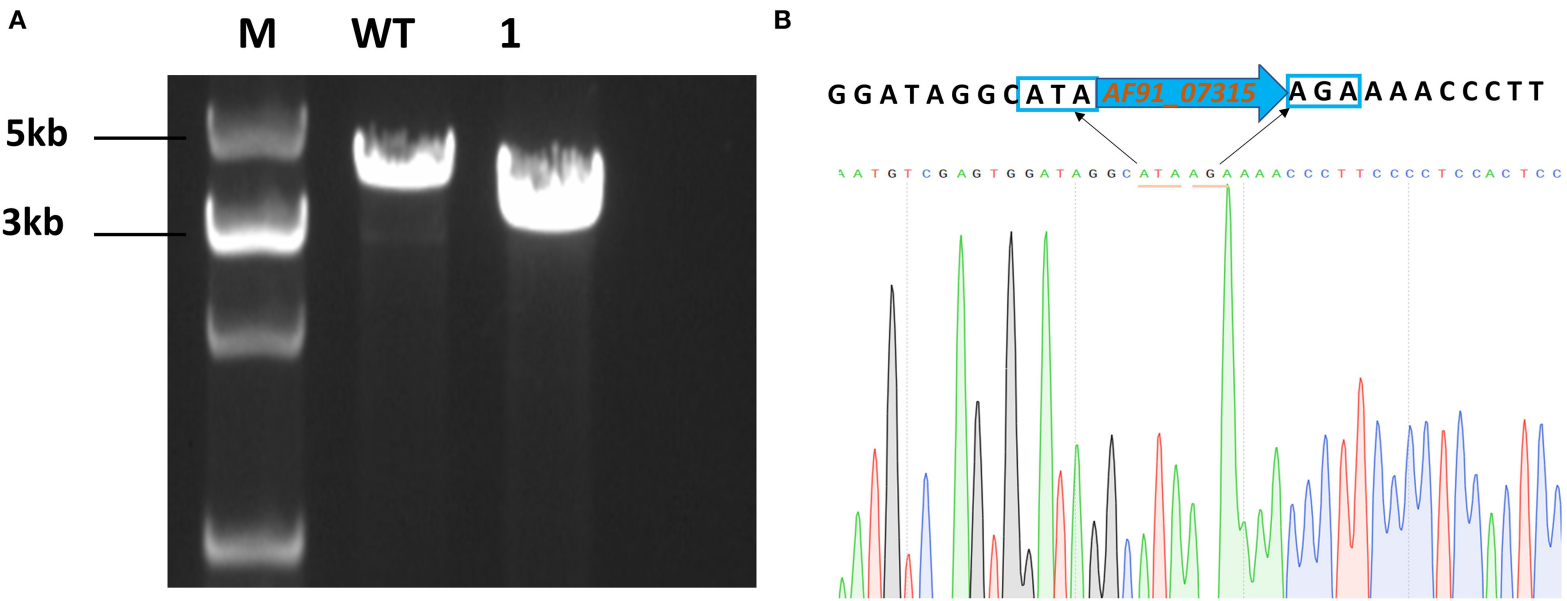
Chromosomal deletion of *ldh* (*AF91_07315*). **(A)** PCR identification of Δ*AF91_07315* mutants. M:5000 bp DNA marker; WT: wild-type strain was used as control (3735bp); lane 1: a mutant strain of *AF91_07315* (2844bp). **(B)** Sequencing of Δ*AF91_07315* mutant.

To produce mutant strains, cells were transformed with pLL-07315. After obtaining a single-clone strain, specific primers flanking the homologous arms were designed for verification using PCR (Figure 1A). The results are shown in Figure 1. The sequencing results further confirmed the gene deletion (Figure 1B).

### 3.2. Fermented milk characteristics of *L. paracasei* CGMCC4691 and mutants

**Figure 2:**
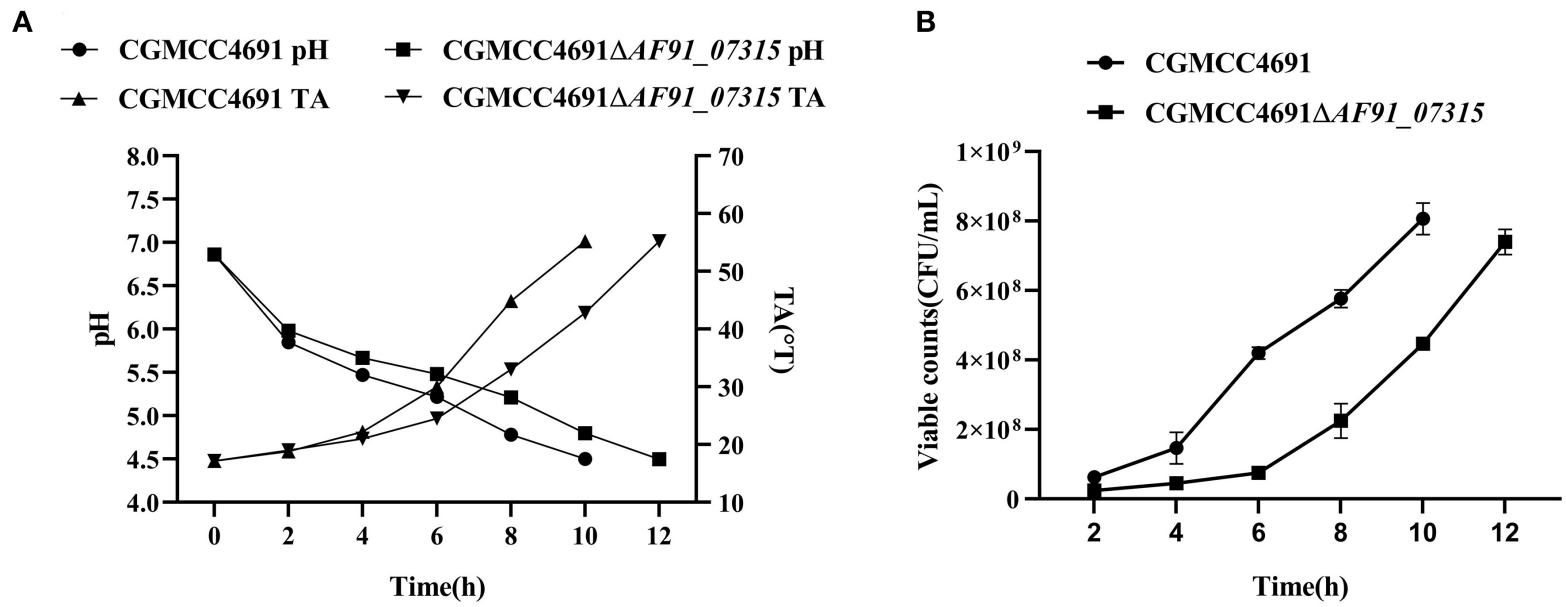
Summary of fermentation characteristics; pH, titration acidity **(A)**, and viable counts **(B)** of *L. paracasei* CGMCC4691 and *L. paracasei* CGMCC4691 Δ*AF91_07315*. The average was calculated from three independent biological replication. The error bars represent the standard deviation.

The pH, TA, and viable counts in the fermented milk were measured every 2 h until the pH reached 4.5. The results are shown in Figure 2. The pH and TA showed opposite trends, and differences were observed as pH decreased (Figure 2A). At 8 h of fermentation, the pH of the mutant strain was 0.4 points higher than the WT strain. Simultaneously, the TA of the mutant strain increased to 33 °T, which was 12 °T higher than that of the WT strain. The fermentation times of the WT and mutant strains were 10 h and 12 h, respectively, at the fermentation end-point. The viable counts consistently decreased during fermentation, and the growth of the WT and mutant strains reached the exponential phase at 4 h (6.4 × 10^7^ CFU/ml) and 6 h (7.5 × 10^7^ CFU/ml), respectively. Next, samples were collected for metabolomic analysis.

### 3.3. Untargeted metabolic analysis of *L. paracasei* CGMCC4691 and *L. paracasei* CGMCC4691Δ*AF91_07315*

#### 3.3.1. Differential metabolic analysis of fermented milk at pre-fermentation

**Figure 3:**
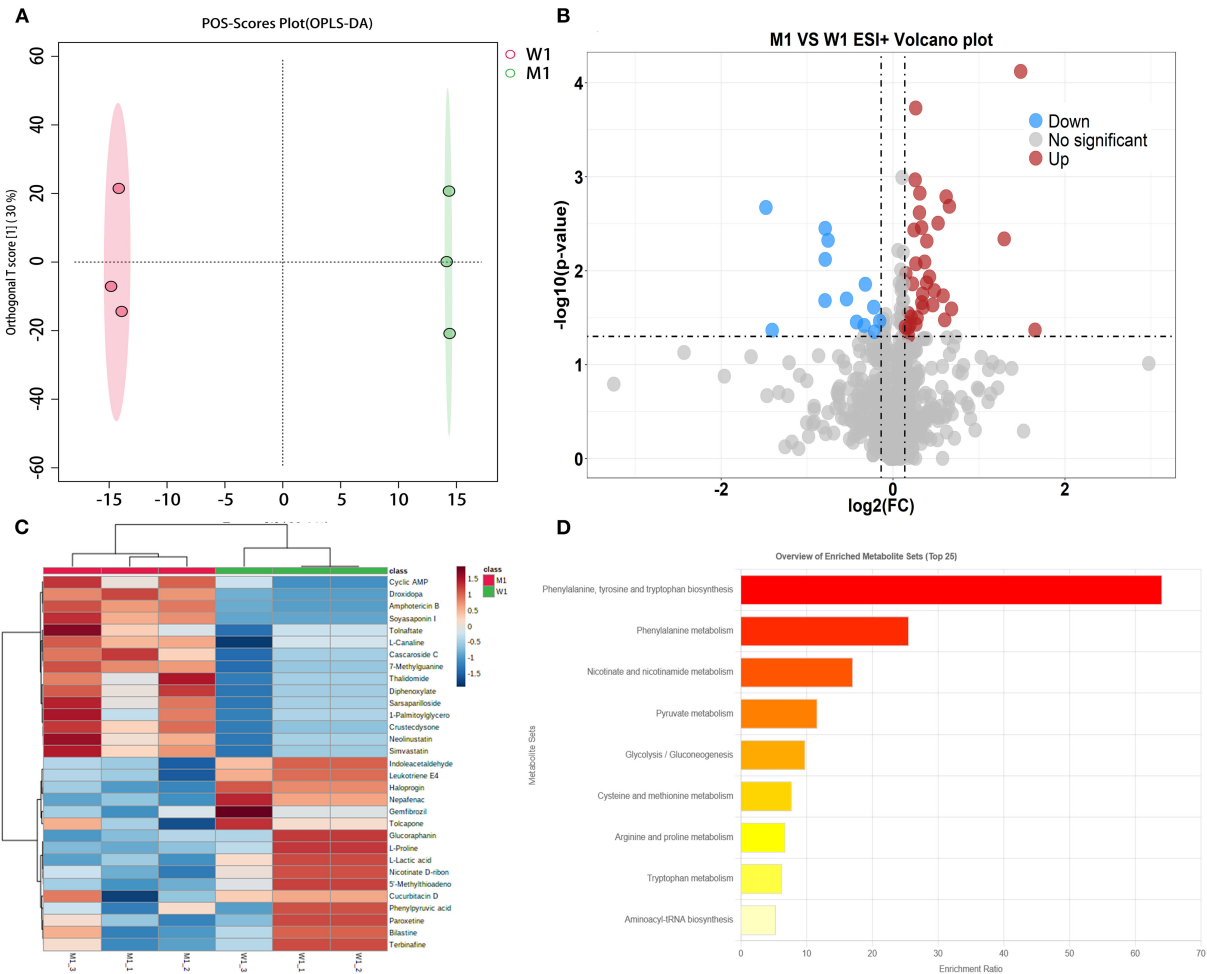
Fermented milk metabolomic analysis of wild-type strain CGMCC4691 (W1) and its mutant strain Δ*AF91_07315* (M1) at 4 h. **(A)** Score plot of orthogonal partial least squares discriminant analysis (OPLS-DA). **(B)** Volcano plot showing differential metabolites in comparison with W1 in the positive ion (ESI+) model, where each dot represents a substance. The abscissa and ordinate indicate fold change (FC) and *p*-value, respectively, after data processing. **(C)** Heatmap indicates the relative abundance of differential metabolites between the two sample groups. **(D)** Pathway enrichment analysis of differential metabolites. The x-axis indicates metabolic pathway impact values obtained from topological analysis and the color scale represents the contribution of metabolites in the pathway. Red indicates the highest and yellow indicates the lowest.

OPLS-DA, a common classification method, was performed to identify the differences between the two groups, which exploits dimension reduction by combining regression models; the regression results were analyzed using a discriminant threshold. A total of 1,096 and 927 substances were generated from the fermented milk in the ESI+ and ESI– modes, respectively. The OPLS-DA of fermented milk is shown in Figure 3. The abscissa represents the principal component score during the quadrature signal correlation; the farther the location of the two milk samples, the greater the difference between the two groups. The ordinate represents the quadrature component score value; the farther the parallel samples are, the greater the difference within the group. The milk fermented by the WT strain at 4 h (W1) was located away from the milk fermented by the mutant strain (M1), indicating that the two groups had significant differences, and a total of 52.4% was explained by the dataset. The critical parameters used to evaluate the OPLS-DA model are presented in Table 2. In the ESI+ model, the R^2^X, R^2^Y, and Q^2^ values were 0.524, 1, and 0.654, respectively. In the ESI– model, the R^2^X, R^2^Y, and Q^2^ values were 0.632, 0.999, and 0.762, respectively, where R^2^ and Q^2^ represent the explanatory ratio and predictability, respectively. The closer the value is to 1, the higher the model accuracy. Generally, when R^2^ and Q^2^ >0.5, the model fits well.

**Table 2:** Critical parameters for evaluating the OPLS-DA model in the positive and negative modes.

**Mode and Sample pair**	**Statistics analysis mode**	**R2X(cum) (cumulative)**	**R2Y(cum) (cumulative)**	**Q2(cum) (cumulative)**
**Positive ion mode**				
M1VSW1	OPLS-DA	0.524	1	0.654
M2VSW2	OPLS-DA	0.806	1	0.896
M3VSW3	OPLS-DA	0.592	0.997	0.759
**Negative ion mode**				
M1VSW1	OPLS-DA	0.632	0.999	0.762
M2VSW2	OPLS-DA	0.541	0.994	0.837
M3VSW3	OPLS-DA	0.584	0.996	0.73

To select the differential metabolites, a volcano plot was generated from the OPLA-DA filtering results (Figure 3B). In ESI+, the red dots indicate that the metabolites were upregulated, with FC >1.1 and *P* ≤ 0.05, and the blue dots indicate that the metabolites were downregulated, with FC <0.9 and *P* ≤ 0.05. The farther the dot is from the origin, the greater the difference between the two groups. A total of 51 metabolites were identified. Next, these potentially differential compounds were further used for enrichment analysis by (1) KEGG ID conversion using the MBRole online database and (2) importing the ID into the 4,691 KEGG metabolic pathways for pathway enrichment analysis. This resulted in 31 compounds, and a heatmap was generated to show their relative abundances (Figure 3C). Enrichment analysis of the two groups was performed using L. paracasei CGMCC4691 (lpq), and nine differential metabolites were enriched in the pathways. The results are shown in Figure 3D, and the metabolites are presented in Table 3. The key pathways include phenylalanine, tyrosine, and tryptophan biosynthesis, as well as phenylalanine metabolism, pyruvate metabolism, and nicotinate and nicotinamide metabolism.

**Table 3:** Differential metabolites identified at the initial stage of fermentation compared with the wild-type strain.

**Name**	**FC**	**vipV**	***p*-value**	**Formula**	**m/z**	**RT**	**KEGG id**
Cucurbitacin D	0.83507	1.879249	0.004019	C30H44O7	515.2997	10.12569	C08796
Cyclic AMP	1.8493	1.651685	0.045625	C10H12N5O6P	328.0485	1.135302	C00575
Indoleacetaldehyde	0.64864	1.779966	0.017819	C10H9NO	158.0612	4.754723	C00637
L-Lactic acid	0.78275	1.905832	0.002613	C3H6O3	89.0249	0.980151	C00186
Leukotriene E4	0.72455	1.714153	0.02395	C23H37NO5S	438.2444	7.451532	C05952
Nicotinate D-ribonucleoside	0.80047	1.711194	0.025026	C11H14NO6	255.0804	14.01767	C05841
Phenylpyruvic acid	0.82577	1.658416	0.044803	C9H8O3	163.0411	2.629356	C00166
5′-Methylthioadenosine	0.59283	2.001216	0.004753	C11H15N5O3S	298.0974	2.392717	C00170
L-Proline	0.8568	1.847439	0.024406	C5H9NO2	116.0708	0.731764	C00148

#### 3.3.2. Differential metabolic analysis of fermented milk at mid-fermentation

**Figure 4:**
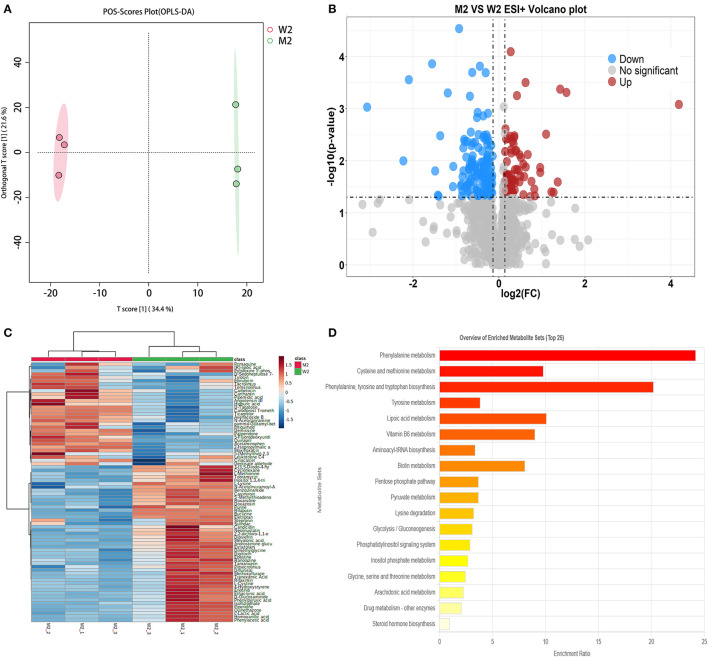
Fermented milk metabolomic analysis of wild-type strain CGMCC4691 (W2) and its mutant strain Δ*ldh* (M2) at 8 h. **(A)** Score plot of orthogonal partial least squares discriminant analysis (OPLS-DA). **(B)** Volcano plot showing differential metabolites in comparison with W1 in the positive ion (ESI+) model, where each dot represents a substance. The abscissa and ordinate indicate fold change (FC) and p-value, respectively, after data processing. **(C)** Heatmap represents the relative abundance of differential metabolites between the two sample groups. **(D)** Pathway enrichment analysis of differential metabolites. The x-axis represents the metabolic pathway impact values obtained from topological analysis, and the color scale represents the contribution of metabolites in the pathway. Red indicates the highest and yellow indicates the lowest.

A method similar to that described above was used to identify differential metabolites between the two sample groups (M2 and W2) at 8 h. In total, 1,117 differential metabolites were identified as potential candidates (VIP >1) in the OPLS-DA plot, and significant separation was observed in the positive mode (Figure 4A). The model evaluation parameters (R^2^X, R^2^Y, and Q^2^ were 0.806, 1, and 0.896 for ESI+; and 0.541, 0.994, and 0.837 for ESI–) indicated that the model was both reliable and valid. In ESI+, the volcano plot showing the FC of these candidates is presented in Figure 4B, and the criteria were FC >1.1 or ≤0.9 and *P* ≤ 0.05. We obtained 207 differential substances from the positive model. The dot represents a variable, and the farther the variable is from the origin, the greater the contribution to the difference between the two groups. Subsequently, the KEGG ID of 80 variables was obtained from the MBRole database, and a heatmap was generated to visualize these substances (Figure 4C). Pathway enrichment analysis was performed using these metabolites (Table 4). A total of 18 signaling pathways are shown in Figure 4D, and the top signaling pathways were phenylalanine metabolism, cysteine and methionine metabolism, biotin metabolism, aminoacyl-tRNA biosynthesis, and pyruvate metabolism.

**Table 4:** Differential metabolites identified at the middle stage of fermentation compared with the wild-type strain.

**Name**	**FC**	**vipV**	***P*-value**	**Formula**	**RT**	**KEGG id**
5′-Methylthioadenosine	0.78193	1.551246	0.012062	C11H15N5O3S	2.392717	C00170
Androsterone glucuronide	0.6413	1.603995	0.005122	C25H38O8	4.40443	C11135
Cyclohexane	0.84815	1.419903	0.038262	C6H12	7.580534	C11249
D-Sedoheptulose 7-phosphate	1.1823	1.406733	0.040502	C7H15O10P	0.961874	C05382
gamma-Glutamyl-beta-aminopropiononitrile	1.2721	1.484472	0.023982	C8H13N3O3	1.022341	C06114
L-Lysine	0.6544	1.580373	0.008054	C6H14N2O2	0.557608	C00047
L-Methionine	0.87224	1.393502	0.044758	C5H11NO2S	0.97255	C00073
Leukotriene C4	1.2231	1.50636	0.018387	C30H47N3O9S	2.463467	C02166
Mevalonic acid	0.68071	1.552892	0.011932	C6H12O4	1.145254	C00418
Primaquine	1.1073	1.433174	0.039787	C15H21N3O	4.431745	C07627
Tobramycin	0.63517	1.627186	0.002817	C18H37N5O9	4.399161	C00397
Tylosin	1.2211	1.488243	0.023878	C46H77NO17	5.1029	C01457
(R)-lipoic acid	1.148	1.425164	0.043946	C8H14O2S2	1.495122	C16241
4-Hydroxystyrene	0.74018	1.401187	0.038492	C8H8O	4.483189	C05627
5-(Methylthio)-2,3-dioxopentyl phosphate	1.1983	1.428032	0.044147	C6H11O6PS	3.779332	C15650
Gentisate aldehyde	1.255	1.515439	0.018559	C7H6O3	3.677932	C05585
Hippuric acid	2.3633	1.414017	0.043724	C9H9NO3	3.423774	C01586
Inositol 1,3,4-trisphosphate	0.68773	1.589626	0.002927	C6H15O15P3	0.530975	C01243
L-Cystine	0.64625	1.450834	0.025939	C6H12N2O4S2	2.63662	C00491
L-Lactic acid	0.8042	1.483528	0.022785	C3H6O3	0.980151	C00186
Moxifloxacin	1.5457	1.663139	0.000941	C21H24FN3O4	10.50109	C07663
Phenylacetic acid	0.62279	1.541756	0.009869	C8H8O2	2.629517	C07086
Phenylpyruvic acid	0.65179	1.473979	0.02142	C9H8O3	2.629356	C00166
Pyridoxine 5′-phosphate	1.2979	1.496891	0.035118	C8H12NO6P	1.824106	C00627

#### 3.3.3. Differential metabolic analysis of fermented milk at the end of fermentation

**Figure 5:**
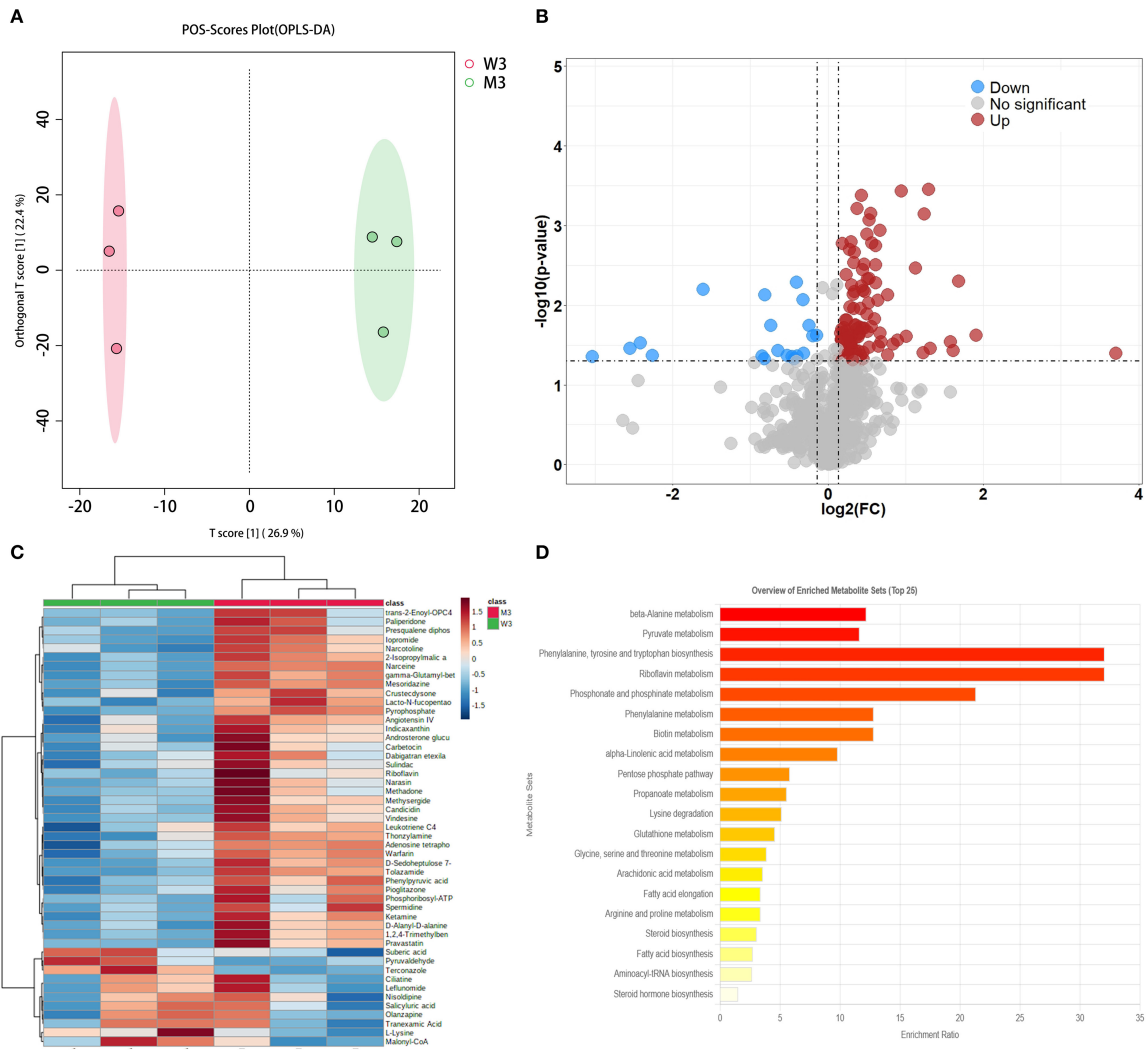
Fermented milk metabolomic analysis of wild-type strain CGMCC4691 (W3) and its mutant strain Δ*ldh* (M3) at the end of the fermentation. **(A)** Score plot of orthogonal partial least squares discriminant analysis (OPLS-DA). **(B)** Volcano plot showing differential metabolites in comparison with W1 in the positive ion (ESI+) model, where each dot represents a substance. The abscissa and ordinate indicate fold change (FC) and *p*-value, respectively, after data processing. **(C)** Heatmap represents the relative abundance of differential metabolites between the two sample groups. **(D)** Pathway enrichment analysis of differential metabolites. The x-axis indicates the metabolic pathway impact values obtained from topological analysis, and the color scale represents the contribution of metabolites in the pathway. Red indicates the highest and yellow indicates the lowest.

The differential metabolites in fermented milk at pH 4.5 were compared using the multiple analyses described above (Figure 5). A total of 1,132 were identified, and OPLS-DA showed significant separation in the positive mode (Figure 5A). The model evaluation parameters (R^2^X, R^2^Y, and Q^2^ = 0.592, 0.997, and 0.759, respectively, in ESI+; and 0.584, 0.996, and 0.73, respectively, in ESI–) are presented in Table 2, which indicates that the model has good stability and predictability. In ESI+, the volcano plot showed 123 differential metabolites according to the criteria mentioned above, where the red and blue dots represent significantly differential metabolites (Figure 5B). Next, **two** statistical analysis methods were performed to further select featured substances: 49 KEGG IDs were obtained from the MBRole database, and a heatmap was used to visualize their relative abundance (Figure 5C). Pathway enrichment analysis was performed using these metabolites, and the details are presented in Table 5. A total of 20 signaling pathways are presented in Figure 5D, and phenylalanine, tyrosine, and tryptophan biosynthesis, as well as biotin metabolism and pyruvate metabolism were involved in key metabolic pathways.

**Table 5:** Differential metabolites identified at the end of fermentation compared with the wild-type strain.

**Name**	**Formula**	**m/z**	**FC**	**VIP**	***p*-value**	**Output**
Androsterone glucuronide	C25H38O8	489.2373	1.669046	1.669046	0.024102	C11135
D-Alanyl-D-alanine	C6H12N2O3	161.0924	1.839027	1.839027	0.004139	C00993
D-Sedoheptulose 7-phosphate	C7H15O10P	291.0487	1.890057	1.890057	0.000617	C05382
gamma-Glutamyl-beta-aminopropiononitrile	C8H13N3O3	200.1035	1.898383	1.898383	0.00071	C06114
L-Lysine	C6H14N2O2	147.1131	1.634573	1.634573	0.040164	C00047
Leukotriene C4	C30H47N3O9S	626.3108	1.567769	1.567769	0.049884	C02166
Malonyl-CoA	C24H38N7O19P3S	854.1408	1.661685	1.661685	0.024537	C00083
Narcotoline	C21H21NO7	400.1322	1.610517	1.610517	0.03951	C09593
Phenylpyruvic acid	C9H8O3	165.0564	1.702135	1.702135	0.027714	C00166
Phosphoribosyl-ATP	C15H26N5O20P4	743.0135	1.622855	1.622855	0.037154	C02739
Pyruvaldehyde	C3H4O2	73.02846	1.536173	1.536173	0.044004	C00546
Riboflavin	C17H20N4O6	377.1469	1.524313	1.524313	0.048974	C00255
Spermidine	C7H19N3	146.1652	1.664869	1.664869	0.03077	C00315
trans-2-Enoyl-OPC4-CoA	C35H54N7O18P3S	986.2695	1.640686	1.640686	0.024748	C16336
2-Isopropylmalic acid	C7H12O5	175.0614	2.1562	1.834587	0.000927	C02504
Ciliatine	C2H8NO3P	124.0161	0.89907	1.792065	0.006253	C03557
Presqualene diphosphate	C30H52O7P2	585.318	2.2076	1.594077	0.033686	C03428
Pyrophosphate	H4O7P2	176.9372	1.3437	1.654038	0.027718	C00013

## 4. Discussion

### 4.1. Fermented milk serves as a vector for studying genetic functions

Lactic acid bacteria (LAB) mutants can change the flavor and texture of fermented milk, and the potential functions of these genes can be inferred from changes in the properties of fermented milk (Sorensen et al., 2016). Courtin et al. (2002) developed *prtS* and *prtB* mutants of *S. thermophilus* and *L. bulgaricus* and confirmed the importance of both genes for strain growth and acidification. Yamauchi et al. (2019) developed a urease-deficient mutant Δ*ureC* of *S. thermophilus* and confirmed that urease is the essential factor for yogurt acidification. In addition, Xu et al. (2021) inactivated the glutathione transport protein (*Ptrp*) gene and measured the glutathione content on the cell surface to determine the transport function of *Ptrp-2* and *Ptrp-4*. Therefore, it is important to study the fermentation characteristics of Δ*AF91_07315* strains using milk as a carrier. Furthermore, mutant strains affected the production of fermented milk metabolites. Although the *AF91_07315* gene mutants obtained using CRISPR technology cannot be applied to food production, the mutant strains in this study can enable us to understand the key metabolic pathways during dairy product development and provide targets for flavor substance interventions.

### 4.2. Advantages of CRISPR technology

The methods used to generate gene mutants of LAB are classified into natural mutagenesis and genetic engineering. Natural mutagenesis has been applied to *L. bulgaricus and L. helveticus* to improve the quality and taste of production (Moller et al., 2007; Guan et al., 2021), which mainly depends on random mutagenesis and large-scale labor-intensive screening. Although the double-crossover homologous recombination strategy compensates for this deficiency and has been used to produce highly purified lactate by cloning the *ldh* gene (Jin et al., 2016; Okano et al., 2018), this method requires the screening of markers. Furthermore, CRISPR gene editing technology has the advantages of high specificity and ease of operation. Tian et al. (2021) used the CRISPR system to knockout and overexpress the *ldh* gene in *L. paracasei* NCBIO01-M2 and screened L-lactate strains with a yield of 221 g/L at 45°C and 99% optical and chemical purity. Our previous study optimized the CRISPR system for *L. paracasei* CGMCC4691; thus, this is the optimal choice to knockout *ldh* using this technique.

### 4.3. Viable count differences between the Δ*AF91_07315* and WT strains

Yogurt is considered beneficial when the bacterial counts reach 10^7^ CFU/ml (Corcoran et al., 2006). TA and pH directly affect the taste. Therefore, fermentation was completed at a pH of 4.5 to ensure that the fermentation process met commercial criteria. The result showed that the Δ*AF91_07315* strain has a lag in both acidity and viability, which is in agreement with Bleckwedel et al., where the *ldh1* gene (mainly responsible for lactate production) of *Fructobacillus tropaeoli CRL 2034* was inactivated, and cell counts and acidity were lagging (Bleckwedel et al., 2020). Viana et al. (2005) reported that the knockout of *ldh* affected the growth rate of mutants in different sugars, showing a higher final pH (5.2) than the WT strain (4.5). Even though lag growth was observed, the viable counts at the end of fermentation were consistent with the WT strain; hence, it had no effect on the fundamental metabolism throughout life.

### 4.4. Differential metabolite screening of fermented milk at different time periods

Non-volatile metabolite profiles were analyzed using liquid chromatography-MS metabolomics during the fermentation process of *L. paracasei* CGMCC4691, and most compounds were upregulated or downregulated compared to those in the WT strain. Specifically, 31, 80, and 49 differential metabolites were identified in the pre, mid, and end stages of fermentation, respectively. The heatmap shows the relative abundance of differential metabolites, where the mid-term metabolites were higher than the pre-term and end stages of fermentation, suggesting the main role of lactate dehydrogenase in mid-fermentation. After enrichment analysis of the metabolic pathway, 9, 24, and 18 substances were identified; however, other substances were not identified, which could be explained by the selection of a model strain for ID conversion. We used *L. paracasei* ATCC334 as a model strain for metabolic pathway analysis, and some metabolites were not involved in metabolic pathway conversion as separate substances in *L. paracasei* CGMCC4691.

#### 4.4.1. Differential metabolites in pre-fermentation

In total, nine differential metabolites were detected in the mutant strains during pre-fermentation when compared to the WT strain. Figure 3D shows the results of the enrichment analysis. The metabolites involved in pyruvate, amino acid, and pyrimidine metabolism included (S)-lactate, phenylpyruvate, L-proline, 5′-methylthioadenosine, and indole-3-acetaldehyde, all of which were downregulated. (S)-lactate, which is catalyzed by lactate dehydrogenase in the last step of glycolysis, provides energy to cells and is the main substance that adds flavor to fermented milk. The Δ*AF91_07315* strain failed to reach the exponential growth phase during pre-fermentation, which may be related to reduced lactate metabolism, insufficient energy supply, and delayed growth of the strain (Rico et al., 2008; Jingjing et al., 2021). In addition, proline, phenylpyruvate, and indole-3-acetaldehyde were downregulated, and the growth-promoting effect of proline on *Lactobacillus* has been demonstrated (Wang et al., 2021a). Sun et al. (2019) added amino acids, uracil, and other substances during the fermentation of *L. rhamnosus*, which improved the start-up speed. In this study, phenylpyruvic acid, indole-3-acetaldehyde, and 5′-methylthioadenosine as the precursors of phenylalanine, tryptophan, and methionine were less synthesized, which affected the growth of the strain (Shapiro, 1962; Yonezawa et al., 2010; Meng et al., 2021).

#### 4.4.2. Differential metabolites in mid-fermentation

During mid-fermentation, the largest gap in pH and viability was observed when compared to the WT strain, and the metabolites detected at this time were mainly L-methionine, 5′-methylthioadenosine, L-cysteine, phenylpyruvate, phenylacetic acid, L-lysine, and L-lactic acid. In particular, the gap in lactic acid content increased compared to the previous stage, which indicates the major role of lactic acid production in regulating the pH of fermented milk (Morelli et al., 1986; Gaspar et al., 2013), and the knockout of *ldh* had a significant effect on lactic acid production. Nevertheless, lactic acid was still detected throughout the fermentation phase in the mutant strain, probably owing to the synergistic effects of other genes encoding acid-producing enzymes (Viana et al., 2005). The synthesis of nutrients, such as amino acids, in LAB is limited to fermented milk; therefore, exogenous nitrogen sources are used to promote their growth, such as the breakdown of amino acids and peptides. In this study, the L-cysteine content was reduced in the Δ*AF91_07315* strain except for methionine, phenylalanine, and its precursors. Wang et al. (2021b) reported different pathways of amino acid biosynthesis at different stages of fermentation, and the synthesis of L-cysteine may be due to different protein hydrolase activities.

#### 4.4.3. Differential metabolites at the end of fermentation

Fermentation was stopped at pH 4.5 to identify the various differential metabolite strains and the differential metabolites detected were all upregulated in the Δ*AF91_07315* strain, including malonyl-CoA, L-lysine, phenylpyruvate, riboflavin, alpha-isopropylmalate, and spermidine, which may be due to the prolonged fermentation time of the mutant strain. In addition, alpha-isopropylmalate and spermidine were involved in pyruvate and arginine metabolism, respectively, providing energy and improving the nutritional quality of fermented milk. Malonyl-CoA is involved in fatty acid synthesis and carbon metabolism, which is related to yogurt flavor (Foster, 2012). Riboflavin is a trace organic substance required for growth and metabolism that stimulates the growth of LAB (Sun et al., 2022). The fermented milk of the Δ*AF91_07315* strain did not complete fermentation when the WT strain was fermented at a pH of 4.5, after which the viable counts of the Δ*AF91_07315* strain continued fermentation and reached the same pH as the WT strain, probably because riboflavin, L-lysine, alpha-isopropylmalate, and spermidine compensated for the growth defect in the Δ*AF91_07315* strain. Further investigation is required because the metabolites of the mutants were upregulated at the end of fermentation, some of which may be toxic, and it is unclear whether there are biosafety issues with fermented milk.

In this study, the *ldh* gene of *L. paracasei* CGMCC4691 was knocked out using CRISPR gene editing technology. The fermented milk results showed that the Δ*AF91_07315* strain had slower growth and acidification capacities than the WT strain. At the initial stage of fermentation, the WT strain entered the exponential growth phase, and metabolites, including (S)-lactate, phenylpyruvate, L-proline, and indole-3-acetaldehyde, were downregulated compared to the WT strain, which affected the start-up speed of the Δ*AF91_07315* strain. At the mid-stage of fermentation, pH and viable counts were the most disparate, with (S)-lactate being the main cause of the differences in pH. In addition, L-cysteine and precursor metabolites involved in amino acid metabolism were reduced because of the influence of protease activity. Although the fermentation time of the mutant strain was prolonged at the end of fermentation, malonyl-CoA, L-lysine, the amino acid intermediate metabolites, phenylpyruvate, and spermidine were upregulated, contributing to the formation of flavored substances. Upregulation of metabolites, such as riboflavin, compensated for the growth defects of the mutant strain. In conclusion, lactate dehydrogenase plays an important role in the growth and acidification of *L. paracasei* and affects the production of flavored milk substances.

## Data availability statement

The original contributions presented in the study are included in the article/supplementary material, further inquiries can be directed to the corresponding author/s.

## Author contributions

XS and SW designed this project and revised the article. SF and LC processed the samples and performed the experiments. SF wrote the article. SF and JB collected the data. HL analyzed the data. SW guided the experiment and provided funding for the research. All the authors read and approved the final submission.
